# Relative Age Effects and contextual factors in male Spanish youth football: a 10-year cross sectional analysis of U12 to U16 players

**DOI:** 10.3389/fspor.2025.1524972

**Published:** 2025-02-27

**Authors:** Iván Peña-González, Gonzalo Fernández-Jávega, Ismael Castellano-Galvañ, Manuel Moya-Ramón

**Affiliations:** Sport Research Centre, Department of Sport Sciences, Miguel Hernández University of Elche, Alicante, Spain

**Keywords:** soccer, maturation, team sports, physical performance, sports performance

## Abstract

**Introduction:**

The Relative Age Effects (RAEs) are complex, multifactorial phenomena influenced by individual (e.g., maturity status), task-related (e.g., field position or competitive level), and environmental (e.g., coaches' expectations) factors. This cross-sectional study aimed to investigate the relationship between RAEs and the maturity status, field position, competitive level and coaches' expectations within a sample of 1,120 young male Spanish football players [mean age: 13.72 ± 1.40 years; weight: 54.09 ± 11.85 kg; height: 162.11 ± 11.38 cm; years from peak height velocity (PHV): −0.22 ± 1.44], across tree age categories (U12, U14 and U16).

**Methods:**

Data was collected over 10 years (2014–2024), considering the maturity status, estimated using the Mirwald et al. (2002) formula. Physical performance was assessed through tests for strength (1RM), power, speed (30-m sprint), agility (*T*-test), jumping (CMJ), and aerobic endurance (estimated VO_2max_). Players' field positions and coaches' efficacy expectations about their players were also collected.

**Results:**

A Chi-square (*χ*²) analysis revealed a skewed distribution across birth quartiles within age categories (*p* < 0.05). Pearsons' correlation and linear regression analyses showed significant relationship between relative age and maturity status (*r* = 0.91; *R*² = 0.84). The RAEs were more pronounced at higher competitive levels, while the distribution bias in playing positions was comparable to the overall sample, with the exception of goalkeepers in the U12-14 categories. ANOVA results tend to a higher physical performance and coaches' efficacy expectations for players with higher RA in the U14 and U16 categories.

**Conclusion:**

This study confirms the presence of strong RAEs over the past decade in youth football players from U12 to U16. Individual and environmental factors, such as advanced maturity status, the intensified selection processes at higher competitive levels, an increased physical performance and higher coaches' efficacy expectations, may contribute to RAEs in a complex and interdependent manner.

## 1 Introduction

The practice of organizing athletes into annual age categories is a widely adopted approach in youth sports, designed to mitigate disparities in physical and cognitive development throughout childhood and adolescence (1). By grouping athletes based on their birth year, this method aims to create a more level playing field by aligning athletes of similar chronological ages. However, this age-based organization inadvertently introduces structural disadvantages known as Relative Age Effects (RAEs) (2). The term relative age (RA) refers to the differences in chronological age among young players born in different months of the same year (3). For instance, a player born in January and another born in December of the same year have an RA difference of nearly twelve months. The consequences of these RA differences among players within the same selection year are referred to as RAEs, which often leads to an overrepresentation of athletes born in the initial months of the year in sports (1, 2, 4). RAEs have been extensively documented across multiple sports, with particularly high prevalence in sports like ice hockey, basketball, and football (soccer), where physical size and strength play a significant role in performance (1).

The impact of RAEs is multifaceted, as player selection processes must consider various factors, including physical, technical, and tactical performance (among others), which influence the likelihood of selection and progression in competitive sports. RAEs may vary not only across different sports or disciplines but also by gender, field positions and competitive levels in which players participate (1, 5). According to Wattie et al. (2), the factors influencing RAEs can be categorized as (1) individual, meaning those related to the person; (2) task-related, referring to the specific roles and demands of the sport; and (3) environmental, pertaining to external factors beyond the athlete. Among the individual factors that may influence RAEs, one example is the athlete's maturity status. The RAEs in sports, and specifically in football, have been suggested to be primarily linked to differences in physiological growth and maturation among young athletes (6), as those born earlier in the year tend to exhibit physical advantages, which in turn can be misinterpreted as indicators of talent (7). These developmental differences can perpetuate a biased view of players' athletic potential, especially in high-contact and high-competition sports like football, where relatively older athletes or those with an advanced maturity status are more likely to be favoured in selection processes. Biological maturation refers to the process leading to adulthood, while the term “status” refers to the current stage or level of progress within the maturation process (6, 8). When the maturity status is controlled, the impact of RAEs on physical performance can decrease, suggesting that the observed effect may be due, at least in part, to maturation differences rather than chronological age alone (9, 10). In other words, the maturity status appears to have a greater impact on the physical performance of young players than RA (11, 12). A youth born in the earlier months of the year is more likely to be advanced in his maturation, in line with his higher RA (11), but it is possible to find a relative younger player with an advanced maturity status or a relative older player being less mature than his mates (6). This distinction highlights the need to treat RAEs and biological maturation as interrelated but independent constructs, as the advantages observed in relatively older players (1) may be attributed to their advanced maturity status rather than their RA alone (13), and (2) are temporal, as late matures can achieve the same or even higher performance than their early maturing peers once maturational processes become equalized (6, 14).

Among the task-related factors, the specific demands of each playing position may influence the selection of players with different RA. It has been suggested that the distribution of players across field positions could also be shaped by the RAEs, as coaches may tend to prefer more physically mature athletes for positions that require specific physical skills (4, 5, 15). However, the impact of RA on field position assignment remains a subject of debate, as no conclusive evidence has been found to support differences in RA across various playing positions, or which specific positions are more influenced by the RAEs (4, 15, 16).

The competitive level may be considered another task-related factor influencing RAEs, as the demands of the sport itself can vary depending on the level of competition, increasing the physical, technical, and tactical requirements placed on athletes as the competitive tier or performance category rises. The magnitude of the RAEs can be considerably higher in sports with high physical demands, particularly at the national or Olympic levels, where selection pressure tends to increase the prevalence of this phenomenon (17, 18). Moreover, previous research suggests that this effect is more prominent in higher-level teams, where selection pressure and performance demands are greater, and it tends to be smaller or non-existent in lower-level teams (16, 17, 19, 20). In football specifically, players born in the first quartiles of the year (Q1) are more likely to be selected for higher-level teams compared to those born in later quartiles (Q4), as observed in multiple population-based studies conducted internationally (21–23). However, it seems that the impact of RAEs tends to diminish as players progress to higher age categories, particularly during the transition to senior levels (24). While the RAEs significantly influence short-term performance in youth and junior categories, it often reverses in senior categories, due to factors such as the maturation of athletes, which equalizes the physical and anthropometric advantages of relatively older players; the increasing importance of technical, tactical, strategic, and psychological skills over physical attributes in advanced competition; and the resilience developed by relatively younger players who overcome RAEs-related challenges (6, 24). This phenomenon aligns with the underdog effect, which posits that relatively younger or late-maturing players develop superior psychological and technical skills due to the greater challenges they face during their development (6).

Thirdly, environmental factors such as the age category or coaches' expectations regarding their players' performance may also influence the RAEs. The age category is an environmental factor influencing RAEs, as it represents an externally imposed structure that organizes athletes by age, shaping opportunities for participation, selection, and development within the sport system. It is this structure, which groups young football players into one-year cohorts, that generates the differences in RA mentioned earlier. On the other hand, the role of social agents, such as coaches and parents, has also been identified as an environmental factor reinforcing the RAEs, as they tend to mistakenly associate physical maturity with superior athletic skills. This phenomenon has been partially explained by the Matthew Effect, the Pygmalion Effect, and the Galatea Effect, where expectations and beliefs influence athletes' performance and opportunities (10, 25). In this context, coaches' efficacy expectations have been studied as a mechanism that can consolidate the competitive advantage of players born in the first months of the year or with greater physical maturity, thereby increasing their chances of reaching higher competitive levels. From this perspective, coaches have shown higher efficacy expectations for players born in the early months of the year, even when the maturity status was controlled (10). This suggests that coaches' beliefs may partly contribute to making the RAEs more pronounced and persistent, as they may provide more opportunities to players with higher RA due to a perceived expectation of greater performance (25, 26).

The aim of this study was to analyse the impact of RAEs in a representative sample of young football players (U12, U14, and U16 categories) by examining how environmental factors (e.g., age category or coaches' expectations), task-related factors (e.g., competitive level or field position distribution) and individual factors (e.g., the player's maturity status) influence its manifestation. Additionally, the study aimed to assess the relationship between RAEs and specific physical performance metrics (e.g., speed, endurance, and strength) to quantify how RAEs may affect athletic capabilities. This comprehensive approach seeks to enhance the understanding of the mechanisms underlying RAEs and its implications for talent identification and development in youth football, providing evidence to support equitable sports policies and foster balanced development opportunities for young athletes.

## 2 Materials and methods

### 2.1 Participants

This study utilized data collected over the past 10 years (2014–2024) on various factors potentially associated with the RAEs in young male Spanish football players within the U12 (11.59 ± 0.58 years), U14 (13.27 ± 0.61 years), and U16 (15.16 ± 0.58 years) categories, representing different teams from five youth football academies. Each age category consists of two one-year cohorts. Players within the same age category can be classified as “first-year” (1st) players (the youngest within the category) or “second-year” (2nd) players (the oldest within the category). This categorization structure allows competition between players spanning two age groups. To appreciate the contextual factors, data collected for this study included individual constraints [e.g., maturity status, by the *maturity offset* (−0.22 ± 1.44 years from/to the PHV)], task-related constraints (e.g., field position distribution), and environmental constraints (e.g., competitive level and coaches' efficacy expectations). A total of 1,120 young football players aged 11–16 years, recruited from different teams and academies, were included in the study. Descriptive data for the sample are provided in Table 1. All players, along with their parents or legal guardians, signed an informed consent form detailing the study's purpose, in accordance with the Declaration of Helsinki.

**Table 1:** Descriptive data for the participants.

Variable	U12 (1st)		U12 (2nd)		U14 (1st)		U14 (2nd)		U16 (1st)		U16 (2nd)	
	*n*	M ± SD	*n*	M ± SD	*n*	M ± SD	*n*	M ± SD	*n*	M ± SD	*n*	M ± SD
Height (cm)	40	143.88 ± 4.94	110	147.16 ± 7.29	261	156.40 ± 7.84	275	163.09 ± 8.46	263	169.64 ± 7.87	166	171.83 ± 7.15
Weight (kg)	40	37.67 ± 6.39	110	41.77 ± 6.73	261	47.39 ± 7.92	275	53.52 ± 9.38	263	61.40 ± 9.11	166	66.10 ± 7.95
1RM (kg)	*nd*	*nd*	*nd*	*nd*	85	59.41 ± 16.15	73	72.22 ± 17.59	51	101.22 ± 20. 03	3	102.00 ± 27.51
PPO (W·kg^−1^)	*nd*	*nd*	*nd*	*nd*	85	584.50 ± 178.05	73	728.11 ± 247.60	51	1,030.57 ± 269.122	3	1,097.17 ± 533.04
CMJ (cm)	40	27.32 ± 4.25	109	28.09 ± 4.72	87	28.88 ± 4.91	98	30.85 ± 5.65	109	33.48 ± 4.72	114	36.33 ± 5.02
30-m sprint (s)	26	5.18 ± 0.19	85	5.23 ± 0.34	205	4.91 ± 0.31	229	4.75 ± 0.32	231	4.51 ± 0.26	127	4.41 ± 0.20
*T*-test (s)	26	10.18 ± 0.44	85	10.03 ± 0.67	172	9.41 ± 0.56	169	9.13 ± 0.66	157	8.86 ± 0.53	98	8.73 ± 0.45
EVO2max (ml·kg·min^−1^)	40	43.81 ± 2.56	95	42.74 ± 2.75	135	44.96 ± 2.97	155	45.38 ± 3.30	160	47.23 ± 3.98	116	48.48 ± 5.15

1RM, one repetition maximum; PPO, peak power output; CMJ, counter movement jump; EVO2max, estimated maximal oxygen uptake; and, No Data.

### 2.2 Procedures

To gain a broader understanding of how various factors influence the RAEs in a large sample of young football players, data were collected over a 10-year period (2014–2024) as part of academies regular testing procedures. In light of this, these data were gathered at different periods throughout the season and analysed using a cross-sectional study design. Due to methodological changes in data collection within these academies, the variables gathered each year may have varied. The most commonly collected variables included the players' RA and maturity status (100% of the cases), competitive level (70%), playing position (90%), and physical performance (60%–90%), as well as the coaches' efficacy expectations (60%–80%) regarding their players.

#### 2.2.1 Relative age

The designated “cut-off” date used to organize young football players into 1-year age categories [i.e., U12(1st)] varies by country; in Spain, this date is set for January 1st. To examine RAEs, players were classified into four birth-month quartiles: Q1 (January to March), Q2 (April to June), Q3 (July to September), and Q4 (October to December). In this system, Q1 includes players with the oldest RA in their category, while Q4 includes the youngest.

#### 2.2.2 Maturity status

Anthropometrical data (body weight and height, leg length and sitting height) was measured using a digital body composition monitor (Tanita Bc 601 Ltd., Japan ± 0.1 kg) and a fixed stadiometer (SECA Ltd., Germany ± 0.1 cm). The decimal age (1) was obtained as:

(1) Decimal Age = (Date of valuation—Date of birth)/365,25

Each player's maturity status was determined by estimating the years from/to Peak Height Velocity (PHV) (2), using the formula by Mirwald et al. (27):

(2) Maturity Offset (boys) = −9.236 + [0.0002708 * (Leg length * Sitting height)] + [0.001663 * (Chronological age * Leg length)] + [0.007216 * (Chronological age * Sitting height)] + [0.02292 * (Weight/Height * 100)]

Estimating the number of years before or after PHV (27) is the most widely used approach to assess somatic maturation in the sports field, with over 1,000 citations of this method from 2002–2023 (28). PHV marks a key period of accelerated growth in stature during adolescence, generally occurring around age 14 in boys and 12 in girls.

##### 2.2.2.1 Competitive level

In Spain, there are three competitive levels (CL) for the U14 and U16 age categories, with CL 1 representing the highest level and CL 3 the lowest. For the U12 category, due to the players' young age, no competitive levels are established to avoid creating a promotion/relegation system that could negatively impact the behavioural or psychological development of young players at such an early stage. Of the sample included in the study, 12% of the players belonged to CL1, 44% to CL2, and 44% to CL3.

#### 2.2.3 Field position

Various scientific publications have categorized football players by playing position, using different specific roles or categories. However, because teams may use varying tactical systems (which involve different on-field positions) and because younger age categories tend to involve less specialization in specific roles, this study categorized players based on their field line position: goalkeepers (GK); defenders (DEF), including central and lateral defenders; midfielders (MF), including defensive and offensive central midfielders, and midfielders playing near the flanks; and forwards (FOR), including central forwards, second strikers, and wingers.

#### 2.2.4 Physical performance

*Maximal Strength:* Maximal strength was assessed through a half-squat one-repetition maximum (1RM) estimation by analyzing movement velocity using a linear encoder (T-Force System, Ergotech, Murcia, Spain) with a 3RM load, and with high validity and reliability (*R*^2^ = 0.98; CV = 0.3%) (29).

*Power:* To assess lower-limb power, the estimation of peak power output (PPO) was conducted. Players performed three half-squats at maximal speed using 60% of their one-repetition maximum (1RM) load. Participants were verbally encouraged to exert maximum effort to ensure they achieved the highest possible movement velocity.

*Linear Speed:* Players completed two trials of a 30-meter linear sprint, starting from a stationary position, with times recorded at the 5-meter and 30-meter marks using photoelectric cells (Witty System, Microgate, Bolzano, Italy). Players were encouraged to sprint at their maximum speed. The best attempt (lower time) was recorded for further analysis.

*Change of Direction:* Players performed two trials of a modified *T*-test (30) which incorporated only forward movements to better align with common football actions and omitting the “touching the cone” requirement (31). Completion time for the *T*-test was recorded using a single start-stop photoelectric cell (Witty System, Microgate, Bolzano, Italy) placed at the point where the test starts and finishes. Players were instructed to run at maximal velocity, and the fastest trial was selected for subsequent analysis.

*Jump Capacity:* Players performed two trials of the Countermovement Jump test (CMJ) keeping the hands on the hips throughout the test (32). Jump height was measured using a contact platform (Globus Ergotester®, Italy), with the highest jump recorded for subsequent analysis.

*Aerobic Endurance:* Maximal oxygen uptake (VO_2max_) was estimated using an intermittent endurance test (30–15 IFT) (33), chosen for its higher specificity to the physical demands of the sport. This estimation was calculated using the following formula (3):

(3) Esimated VO_2max_ = 28.3—(2.15 × Gender)—(0.741 × Age)—(0.0357 × Weight) + (0.0586 × Age × vIFT) + (1.03 × vIFT)

The validity and reliability of this method for estimating aerobic capacity using the 30–15 IFT test have been widely demonstrated and have been proposed as appropriate and extremely useful for team sports (34).

#### 2.2.5 Coaches' efficacy expectations

To assess coaches' efficacy expectations, coaches were asked to rate their confidence in each player's capability to perform specific physical performance tests, adhering to Bandura's (35) guidelines and employing the same questionnaire utilized in Peña-González et al. (10). The questionnaire was always administered to the coaches immediately prior to the assessment of the players. An example question presented to the coaches was: “Indicate your level of confidence in your player's performance on an indirect Repetition Maximum test for the squat exercise, a test designed to assess maximal strength in the squat movement,” thereby capturing the coach's expectations regarding the player's performance on this specific test (10, 35). Additionally, coaches were asked to evaluate their confidence in each player's overall football-playing ability. This single item, referred to as Football Performance Expectations (FP-Exp), assesses the coaches' confidence in their players' comprehensive performance, encompassing physical, technical, and tactical skills. Literature supports the use of single-item measures to evaluate both individual and collective efficacy in performance contexts (36). Each item was rated on a Likert scale from 1 (“no confidence”) to 5 (“maximum confidence”). Previous research by Peña-González et al. (10) identified a single factor among items related to physical performance tests, with a Cronbach's alpha of 0.87, indicating good internal reliability. This factor, termed Physical Performance Expectations (PP-Exp), is similarly applied in the present study.

### 2.3 Statistical analisys

The RAEs, as well as the comparison of physical performance and efficacy expectations among players from different Q, was presented by one-year cohorts [e.g., U12(1^st^), U12(2^nd^)]. In contrast, the distribution by competitive levels and specific field positions was shown by age categories (U12, U14, and U16), following the structure of competition at these ages. A Chi-square test (*χ*²) was employed to assess a potential skew in the distribution of players among Q by age categories, CLs, and field positions. The relationship between players' chronological age and maturity status was assessed using a simple linear regression analysis for each age category, where both variables were expressed as continuous variables in decimal format. Pearson's correlation coefficient (*r*) was included to report the relationship between age and maturity status and it was interpreted as: trivial (<0.09), small (0.10–0.29), moderate (0.30–0.49), high (0.50–0.69), very high (0.70–0.89) and almost perfect (>0.90) (37). The coefficient of determination (*R*^2^) of the linear regression was also included to show the common variance between age and maturity status. A one-way analysis of variance (ANOVA) was conducted to examine differences in physical performance and coaches' efficacy expectations across birth quartiles for each age category. Where significant differences were identified, *post-hoc* Bonferroni tests were applied to assess pairwise comparisons. To complement these analyses, effect sizes (ES) between Q1 and Q4 players were calculated using Hedges' g (38), with interpretation as follows: *g* > 0.8 (large effect), 0.5–0.8 (moderate effect), 0.2–0.5 (small effect), and <0.2 (trivial effect). All statistical analyses were performed using custom spreadsheets developed in Microsoft Excel (Microsoft, Seattle, USA) and JASP software (version 0.13, JASP, Amsterdam, Netherlands). Data were analysed with a threshold for statistical significance set at *p* < .05.

## 3 Results

### 3.1 Environmental factors: RA distribution across age categories and coaches' efficacy expectations

**Figure 1:**
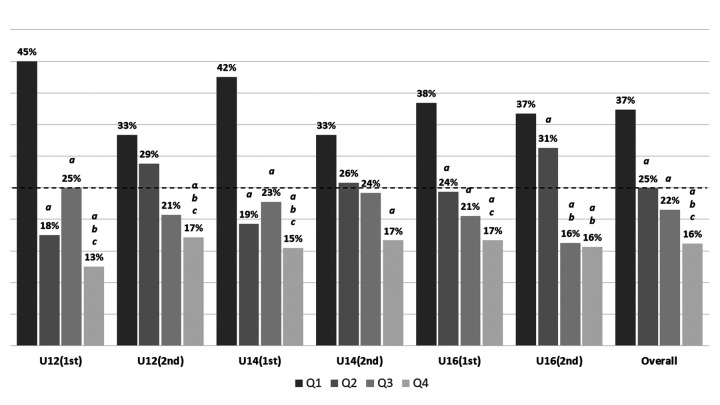
Player's distribution across birth quartiles for each age category and the overall sample. ^a^Significant difference with Q1; ^b^Significant difference with Q2; ^c^Significant difference with Q3. 25% expected distribution.

The *χ*² test revealed a skewed distribution of players born in different Q of the year across the three age categories (Figure 1).

No systematic differences were found in coaches' efficacy expectations for players from different birth quartiles in the U12 category (Table 2). Significant differences were found among Q in PP-Exp (favouring early born players) in U14(1st) and U16(1st), while differences in FP-Exp were found in U14(2nd).

**Table 2:** Coaches' efficacy expectations of U12, U14 and U16 football players across the 4 birth quartiles (Q1, Q2, Q3 and Q4).

Variable	Q1	Q2	Q3	Q4	F *(p)*	ES (Q1-Q4)
U12 (1st)						
CMJ-Exp	3.72 ± 0.75	3.57 ± 0.54	3.50 ± 0.53	3.20 ± 0.84	0.83 (0.49)	0.65 (−0.36; 1.66)
30-m-Exp	3.94 ± 0.80	3.57 ± 0.79	3.80 ± 0.92	3.20 ± 0.84	1.17 (0.34)	0.88 (−0.14; 1.91)
*T*-test-Exp	3.72 ± 0.90	3.86 ± 0.90	3.70 ± 0.95	3.20 ± 0.84	0.58 (0.64)	0.56 (−0.44; 1.57)
EVO_2max_-Exp	3.83 ± 0.86	3.86 ± 1.07	4.00 ± 1.05	3.20 ± 1.10	0.78 (0.51)	0.67 (−0.34; 1.68)
PP-Exp	3.81 ± 0.73	3.71 ± 0.73	3.75 ± 0.73	3.20 ± 0.82	0.91 (0.45)	0.79 (−0.23; 1.80)
FP-Exp	4.44 ± 0.86	4.29 ± 0.95	4.30 ± 0.68	4.40 ± 0.55	0.11 (0.96)	0.05 (−0.94, 1.04)
U12 (2nd)						
CMJ-Exp	3.71 ± 0.93	3.70 ± 0.70	3.47 ± 0.96	3.63 ± 1.07	0.33 (0.80)	0.08 (−0.48; 0.64)
30-m-Exp	3.86 ± 0.88	3.67 ± 0.76	3.53 ± 1.02	3.32 ± 1.16	1.51 (0.22)	0.54 (−0.03, 1.11)
*T*-test-Exp	3.80 ± 0.90	3.63 ± 0.77	3.58 ± 0.96	3.53 ± 0.96	0.50 (0.69)	0.36 (−0.20; 0.92)
EVO_2max_-Exp	3.69 ± 1.11	3.50 ± 0.90	3.42 ± 1.07	3.47 ± 1.12	0.35 (0.79)	0.19 (−0.37; 0.75)
PP-Exp	3.76 ± 0.83	3.63 ± 0.69	3.50 ± 0.89	3.49 ± 0.99	0.64 (0.59)	0.30 (−0.26; 0.86)
FP-Exp	3.66 ± 0.91	3.70 ± 0.75	3.47 ± 1.02	3.58 ± 1.12	0.26 (0.85)	0.08 (−0.48; 0.64)
U14 (1st)						
1RM-Exp	3.79 ± 0.86	3.19 ± 0.87	3.29 ± 1.01	3.25 ± 0.70	2.39 (0.08)	0.64 (−0.16; 1.43)
PPO-Exp	3.90 ± 0.82	3.14 ± 0.73^a^	3.29 ± 1.01	3.38 ± 0.52	4.04 (0.01)*	0.66 (−0.14; 1.46)
CMJ-Exp	3.95 ± 0.88	3.40 ± 0.83	4.00 ± 0.82	3.33 ± 1.21	2.31 (0.08)	0.65 (0.07; 1.23)
30-m-Exp	3.80 ± 0.88	3.56 ± 1.11	3.38 ± 0.81	3.07 ± 1.07	3.25 (0.02)*	0.79 (0.20; 1.38)
*T*-test-Exp	3.99 ± 0.87	3.56 ± 0.81	3.50 ± 0.93^a^	3.14 ± 1.03^a^	5.23 (<0.01)*	0.94 (0.06, 1.82)
EVO_2max_-Exp	3.83 ± 0.93	3.67 ± 0.90	3.79 ± 0.92	3.33 ± 1.21	0.53 (0.67)	0.51 (0–36; 1.37)
PP-Exp	3.88 ± 0.76	3.44 ± 0.75^a^	3.51 ± 0.82	3.21 ± 0.87^a^	4.71 (<0.01)*	0.85 (0.26; 1.44)
FP-Exp	4.15 ± 0.94	3.86 ± 0.80	4.02 ± 0.86	3.79 ± 1.05	1.13 (0.34)	0.37 (−0.21; 0.95)
U14 (2nd)						
1RM-Exp	3.96 ± 1.04	3.56 ± 0.71	3.50 ± 0.79	4.33 ± 0.82	2.19 (0.10)	−0.36 (−1.24; 0.53)
PPO-Exp	4.00 ± 1.02	3.44 ± 0.86	3.67 ± 0.84	4.67 ± 0.82^b^	3.22 (0.03)*	−0.66 (−1.56; 0.24)
CMJ-Exp	3.21 ± 0.79	3.33 ± 1.16	3.82 ± 0.85	3.44 ± 1.03	1.55 (0.21)	−0.25 (−0.92; 0.42)
30-m-Exp	3.85 ± 1.18	3.69 ± 0.95	3.40 ± 1.08	3.59 ± 1.22	1.25 (0.30)	0.22 (−0.29; 0.72)
*T*-test-Exp	3.89 ± 1.03	3.74 ± 1.02	3.58 ± 1.13	3.55 ± 1.10	0.87 (0.46)	0.32 (−0.19; 0.83)
EVO_2max_-Exp	3.32 ± 0.89	3.29 ± 1.06	3.96 ± 0.90	3.19 ± 0.83	2.94 (0.04)*	0.15 (−0.52; 0.81)
PP-Exp	3.78 ± 0.97	3.56 ± 0.80	3.62 ± 0.83	3.60 ± 1.05	0.51 (0.68)	0.18 (−0.33; 0.69)
FP-Exp	4.17 ± 0.96	3.67 ± 1.22	4.05 ± 1.04	3.46 ± 0.96	3.23 (0.02)*	0.73 (0.21; 1.25)
U16 (1st)						
CMJ-Exp	3.39 ± 0.89	3.39 ± 1.04	3.73 ± 0.47	2.69 ± 0.86^c^	3.11 (0.03)*	0.78 (0.07; 1.48)
30-m-Exp	3.53 ± 0.90	0.32 ± 0.95	3.30 ± 0.82	2.86 ± 0.86	2.14 (0.10)	0.74 (0.14; 1.35)
*T*-test-Exp	3.51 ± 0.95	3.44 ± 1.04	3.44 ± 0.51	2.79 ± 0.80^a^	2.55 (0.06)	0.77 (0.17; 1.38)
EVO_2max_-Exp	3.57 ± 0.99	3.44 ± 1.15	3.73 ± 0.65	2.92 ± 1.04	1.57 (0.21)	0.63 (−0.06; 1.33)
PP-Exp	3.53 ± 0.73	3.48 ± 0.82	3.46 ± 0.44	2.86 ± 0.77^a^	3.46 (0.02)*	0.90 (0.28; 1.51)
FP-Exp	3.65 ± 0.69	3.68 ± 0.99	3.44 ± 0.73	3.07 ± 1.39	1.91 (0.13)	0.65 (0.05; 1.25)
U16 (2nd)						
CMJ-Exp	4.19 ± 0.75	3.75 ± 0.87	3.83 ± 0.75	3.86 ± 0.38	0.92 (0.44)	0.48 (−0.42; 1.38)
30-m-Exp	4.06 ± 0.77	3.42 ± 0.90	3.33 ± 1.03	3.71 ± 0.49	1.94 (0.14)	0.48 (−0.42;1.38)
*T*-test-Exp	3.88 ± 0.81	3.33 ± 0.99	3.67 ± 0.82	4.00 ± 0.82	1.22 (0.32)	−0.14 (1–03; 0.75)
EVO_2max_-Exp	3.81 ± 0.91	3.67 ± 0.89	4.00 ± 0.89	3.71 ± 0.49	0.23 (0.88)	0.12 (−0.77; 1.01)
PP-Exp	3.99 ± 0.64	3.54 ± 0.82	3.71 ± 0.83	3.82 ± 0.35	0.98 (0.41)	0.29 (−0.61; 1.18)
FP-Exp	4.13 ± 0.62	4.00 ± 0.85	4.00 ± 0.89	4.00 ± 1.00	0.08 (0.97)	0.17 (−0.72; 1.06)

Q, birth quartile; ES, effect size; Exp, expectations.

^a^ Statistically different from Q1.

^b^ Statistically different from Q2.

^c^ Statistically different from Q3.

* *p* < .05.

### 3.2 Task-related factors: competitive levels and field positions

The *χ*² analysis showed an even greater participation of players born in Q1 and a lower participation of those born in Q4 at the highest competitive level (CL1) (Table 3), using the general distribution of players by birth Q in each age category—already shown to be skewed in favour of Q1 (Figure 1)—as the expected distribution. In CL3, the skewness in birth Q distribution was reduced, though not significantly. Furthermore, there was no consistent increase or decrease in the RAEs by specific positions compared to the initially skewed distribution by category used as the expected distribution, except for the GK position (Table 4). For GKs, a reduction in the RAEs was observed in U12, with a decrease in the percentage of players from Q1 (−6.3%) and an increase in players from Q4 (12.7%), as well as in U14, where an increase in Q4 players was noted (7.5%). This trend did not appear significant in U16 or across the total sample.

**Table 3:** Observed frequencies and increments (in percentage) for each birth quartile (Q) by competitive level (CL), according to the expected distribution.

Competitive level	Q1	Δ	Q2	Δ	Q3	Δ	Q4	Δ
**ED_U14_**	**38**.**3%**		**22**.**7%**		**22**.**5%**		**16**.**4%**	
CL1	41.9%	3.6%	20.9%	−1.8%	27.9%	5.4%	9.3%^a^^,^^b^^,^^c^	−7.1%
CL2	42.6%	4.3%	26.5%	3.8%	20.0%	−2.5%	11.0%	−5.4%
CL3	35.5%	−2.8%	23.7%	1.0%	21.1%	−1.4%	19.7%	3.3%
**ED_U16_**	**37**.**5%**		**26**.**6%**		**19**.**5%**		**16**.**4%**	
CL1	53.3%	15.8%	30.0%^a^	3.4%	10.0%^a^^,^^b^	−9.5%	6.7%^a^^,^^b^^,^^c^	−9.7%
CL2	48.1%	10.6%	22.6%	−4.0%	18.9%	−0.6%	10.4%^a^^,^^b^	−6.0%
CL3	33.3%	−4.2%	24.1%	−2.5%	20.4%	0.9%	22.2%	5.8%
**ED_TOT_**	**37**.**4%**		**24**.**6%**		**21**.**9%**		**16**.**2%**	
CL1	46.6%	9.2%	24.7%	0.1%	20.5%	−1.3%	8.2%^a^^,^^b^^,^^c^	−8.0%
CL2	44.8%	7.5%	24.9%	0.3%	19.5%	−2.4%	10.7%	−5.4%
CL3	34.6%	−2.7%	23.8%	−0.7%	20.8%	−1.1%	20.8%	4.6%

ED, expected distribution; CL, competitive level.

^a^ Statistically different from Q1.

^b^ Statistically different from Q2.

^c^ Statistically different from Q3.

**Table 4:** Observed frequencies and increments (in percentage) for each birth quartile (Q) by field positions (FP), according to the expected distribution for each category.

Field position	Q1	Δ	Q2	Δ	Q3	Δ	Q4	Δ
**ED_U12_**	**34**.**1%**		**25**.**1%**		**25**.**7%**		**15**.**1%**	
GK	27.8%	−6.3%	33.3%	8.2%	11.1%^a^^,^^b^	−14.6%	27.8%^a^^,^^b^^,^^c^	12.7%
DEF	38.3%	4.2%	25.5%	0.4%	19.2%	−6.5%	17.0%	1.9%
MF	34.2%	0.1%	21.1%	−4.1%	31.6%	5.9%	13.2%	−1.9%
FOR	40.4%	6.3%	25.5%	0.4%	21.3%	−4.4%	12.8%	−2.3%
**ED_U14_**	**38**.**3%**		**22**.**7%**		**22**.**5%**		**16**.**4%**	
GK	40.2%	1.9%	18.5%	−4.2%	17.4%	−5.1%	23.9%^b^^,^^c^	7.5%
DEF	34.3%	−4.0%	26.6%	3.9%	23.1%	0.6%	16.1%	−0.3%
MF	42.1%	3.8%	22.4%	−0.3%	24.3%	1.8%	11.2%	−5.2%
FOR	37.7%	−0.6%	23.9%	1.1%	23.1%	0.6%	15.4%	−1.0%
**ED_U16_**	**37**.**5%**		**26**.**6%**		**19**.**5%**		**16**.**4%**	
GK	29.3%	−8.3%	26.8%	0.2%	23.2%	3.7%	20.7%	4.3%
DEF	47.5%	10.0%	22.1%	−4.5%	17.2%	−2.3%	13.1%	−3.3%
MF	34.9%	−2.6%	27.9%	1.3%	25.6%	6.1%	11.6%	−4.8%
FOR	35.0%	−2.6%	31.1%	4.5%	16.5%	−3.0%	17.5%	1.1%
**ED_TOT_**	**37**.**4%**		**24**.**6%**		**21**.**9%**		**16**.**2%**	
GK	34.4%	−3.0%	23.4%	−1.1%	19.3%	−2.6%	22.9%	6.7%
DEF	40.1%	2.7%	24.7%	0.1%	20.2%	−1.7%	15.1%	−1.1%
MF	38.1%	0.7%	24.2%	−0.3%	26.0%	4.1%	11.7%	−4.5%
FOR	37.1%	−0.2%	26.8%	2.2%	20.4%	−1.5%	15.7%	−0.5%

ED, expected distribution; CL, competitive level.

^a^ Statistically different from Q1.

^b^ Statistically different from Q2.

^c^ Statistically different from Q3.

### 3.3 Individual factors: maturity status and physical performance

**Figure 2:**
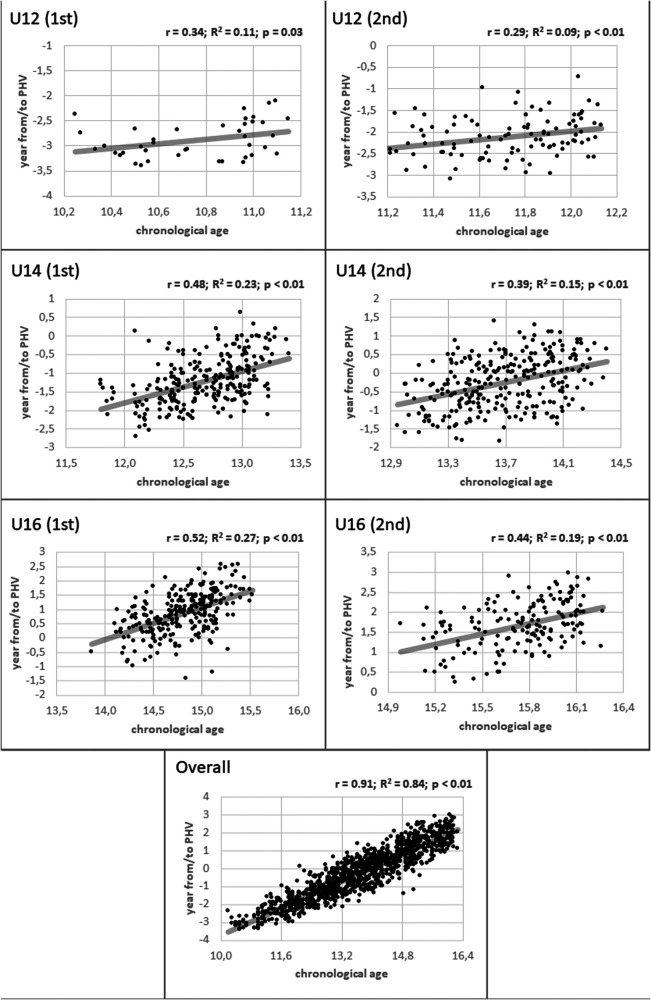
Linear regression analysis of chronological age (expressed in decimal age to reflect relative age) and maturity status by age category. *r*: Pearson's correlation coefficient; *R*^2^: Coefficient of determination.

Pearson's correlation and linear regression analyses showed a significant relationship between age and maturity status for the U12 (*r* = 0.34 and 0.39; *R*^2^ = 0.11 and 0.09; *p* = 0.03 and <0.01, for the 1st and 2nd year of this category, respectively), U14 (*r* = 0.48 and 0.39; *u*^2^ = 0.23 and 0.15; *p* < 0.01) and U16 categories (*r* = 0.52 and 0.44; *R*^2^ = 0.27 and 0.19; *p* < 0.01), as well as for the overall sample (*r* = 0.91; *R*^2^ = 0.84; *p* < 0.01) (Figure 2).

The ANOVA revealed no differences in weight, height, or physical performance variables between players from different quartiles in the U12 category (Table 5). Some differences in anthropometric and physical performance variables between players from different Q in U14 and U16 categories are shown in Table 4. Q1 players were taller in U14 (1st and 2nd) and heavier in U14(1st). Q1 players in U14 were also faster in the 30-m sprint and *T*-test both in 1st and 2nd ages, while they had better results in 1RM, PPO and CMJ only in the U14(2nd). For the U16 category, differences among Q were shown for height and *T*-test in the 1st age of the category, while for weight and 30-m sprint in the 2nd age.

**Table 5:** Anthropometric and physical performance variables of U12, U14 and U16 football players across the 4 birth quartiles (Q1, Q2, Q3 and Q4).

Variable	Q1	Q2	Q3	Q4	F *(p)*	ES (Q1-Q4)
U12 (1st)						
Height (cm)	145.30 ± 5.99	142.46 ± 3.11	142.02 ± 4.54	144.44 ± 1.72	1.20 (*0.32)*	0.15 (−0.84; 1.14)
Weight (kg)	38.41 ± 7.26	35.99 ± 2.36	35.38 ± 4.27	41.92 ± 9.04	1.46 (*0.24)*	−0.44 (−1.44; 0.56)
CMJ (cm)	27.83 ± 3.67	26.34 ± 4.70	27.75 ± 3.85	26.02 ± 6.87	0.38 (*0.77)*	0.39 (−0.61; 1.39)
30-m sprint (s)	5.16 ± 0.20	5.26 ± 0.20	5.13 ± 0.17	5.25 ± 0.23	0.57 (*0.64)*	−0.42 (−1.67; 0.84)
*T*-test (s)	10.09 ± 0.38	10.54 ± 0.61	10.14 ± 0.40	10.14 ± 0.55	1.11 (*0.37)*	−0.12 (−1.36; 1.13)
EVO_2max_ (ml·kg·min^−1^)	44.44 ± 2.79	43.96 ± 3.06	43.88 ± 1.39	41.18 ± 1.48	2.35 (*0.09)*	1.21 (0.16; 2.26)
U12 (2nd)						
Height (cm)	148.61 ± 6.27	146.24 ± 8.76	147.06 ± 6.75	145.95 ± 7.24	0.82 (*0.48)*	0.40 (−0.16; 0.95)
Weight (kg)	41.88 ± 5.54	41.54 ± 8.22	40.80 ± 6.10	43.07 ± 7.17	0.41 (*0.75)*	−0.19 (−0.75; 0.36)
CMJ (cm)	28.82 ± 4.75	28.77 ± 4.10	27.92 ± 4.95	25.77 ± 4.88	2.11 (*0.10)*	0.63 (0.06; 1.19)
30-m sprint (s)	5.18 ± 0.31	5.17 ± 0.30	5.28 ± 0.27	5.36 ± 0.45	1.49 (*0.22)*	−0.49 (−1.10; 0.12)
*T*-test (s)	9.98 ± 0.66	9.93 ± 0.59	9.93 ± 0.72	10.36 ± 0.71	1.69 (*0.18)*	−0.55 (−1.17; 0.06)
EVO_2max_ (ml·kg·min^−1^)	43.04 ± 2.98	42.87 ± 1.88	42.39 ± 2.74	42.44 ± 3.41	0.33 (*0.81)*	0.19 (−0.41; 0.78)
U14 (1st)						
Height (cm)	159.28 ± 7.62	154.81 ± 7.21^a^	154.93 ± 7.43^a^	153.35 ± 7.69^a^	8.87 (<0.01)*	0.77 (0.39; 1.14)
Weight (kg)	49.56 ± 7.99	46.79 ± 8.15	46.12 ± 6.89^a^	44.55 ± 7.84^a^	5.17 (<0.01)*	0.62 (0.25; 1.00)
1RM (kg)	62.37 ± 17.42	59.29 ± 16.58	56.20 ± 12.17	55.33 ± 18.13	0.84 (*0.48)*	0.39 (−0.34; 1.13)
PPO (W·kg^−1^)	603.62 ± 172.51	566.64 ± 189.83	591.80 ± 175.94	535.62 ± 193.67	0.43 (*0.73)*	0.38 (−0.36; 1.11)
CMJ (cm)	29.57 ± 5.49	29.50 ± 4.19	28.09 ± 3.74	26.18 ± 4.90	1.46 (*0.23)*	0.62 (−0.11; 1.35)
30-m sprint (s)	4.81 ± 0.29	4.89 ± 0.28	5.00 ± 0.27^a^	5.03 ± 0.37^a^	6.37 (<0.01)*	−0.69 (−1.12; −0.26)
*T*-test (s)	9.30 ± 0.53	9.35 ± 0.51	9.55 ± 0.53	9.68 ± 0.73^a^	3.58 (0.02)*	−0.66 (−1.18; −0.14)
EVO_2max_ (ml·kg·min^−1^)	44.97 ± 2.92	45.18 ± 3.69	44.89 ± 2.94	44.83 ± 2.68	0.06 (*0.98)*	0.05 (−0.42; 0.52)
U14 (2nd)						
Height (cm)	165.04 ± 8.85	162.99 ± 8.57	161.41 ± 7.55	161.53 ± 8.03	3.09 (0.03)*	0.40 (0.05; 0.75)
Weight (kg)	54.88 ± 9.19	53.10 ± 9.70	52.30 ± 8.87	53.05 ± 9.86	1.09 (*0.35)*	0.19 (−0.16; 0.54)
1RM (kg)	74.48 ± 19.90	73.68 ± 14.72	66.37 ± 13.68	75. 17 ± 24.57	0.96 (*0.96)*	−0.03 (−0.91; 0.85)
PPO (W·kg^−1^)	797.66 ± 253.52	665.34 ± 261.77	638.05 ± 141.88	876.85 ± 321.94	2.94 (0.04)*	−0.29 (−1.17; 0.59)
CMJ (cm)	32.63 ± 4.99	31.04 ± 5.64	31.25 ± 6.33	27.52 ± 4.46^a^	3.15 (0.03)*	1.05 (0.40; 1.70)
30-m sprint (s)	4.65 ± 0.30	4.77 ± 0.29	4.77 ± 0.31	4.88 ± 0.37^a^	5.13 (<0.01)*	−0.70 (−1.10; −0.30)
*T*-test (s)	8.93 ± 0.55	9.08 ± 0.65	9.17 ± 0.59	9.62 ± 0.82^a^^,^^b^^,^^c^	6.73 (<0.01)*	−1.06 (−1.57; −0.55)
EVO_2max_ (ml·kg·min^−1^)	46.24 ± 3.24	45.27 ± 3.00	44.67 ± 3.24	44.91 ± 3.78	1.87 (*0.14)*	0.38 (−0.09; 0.85)
U16 (1st)						
Height (cm)	171.95 ± 7.65	168.58 ± 7.49^a^	168.84 ± 7.66	166.86 ± 7.99^a^	5.58 (<0.01)*	0.65 (0.29; 1.01)
Weight (kg)	62.70 ± 8.67	61.04 ± 9.33	60.65 ± 9.10	59.83 ± 9.68	1.27 (*0.28)*	0.32 (−0.04; 0.67)
CMJ (cm)	33.60 ± 4.20	33.92 ± 4.89	34.14 ± 6.19	31.97 ± 4.33	0.84 (*0.47)*	0.38 (−0.16; 0.92)
30-m sprint (s)	4.48 ± 0.22	4.51 ± 0.26	4.49 ± 0.28	4.59 ± 0.28	1.94 (*0.12)*	−0.45 (−0.83; −0.07)
*T*-test (s)	8.70 ± 0.52	8.92 ± 0.47	8.84 ± 0.50	9.37 ± 0.38^a^^,^^b^^,^^c^	9.94 (<0.01)*	−1.34 (−1.87; −0.81)
EVO_2max_ (ml·kg·min^−1^)	47.59 ± 4.45	47.56 ± 3.80	47.25 ± 3.34	46.21 ± 3.93	0.99 (*0.40)*	0.32 (−0.11; 0.75)
U16 (2nd)						
Height (cm)	173.13 ± 6.54	171.59 ± 7.16	171.63 ± 9.54	169.49 ± 5.04	1.65 (*0.18)*	0.59 (0.12; 1.05)
Weight (kg)	67.36 ± 8.06	67.06 ± 8.05	65.37 ± 7.85	61.95 ± 6.40^a^^,^^b^	3.34 (0.02)*	0.70 (0.23; 1.17)
CMJ (cm)	37.89 ± 4.70	35.40 ± 5.31	36.30 ± 5.56	35.11 ± 4.23	2.13 (*0.10)*	0.61 (0.08; 1.13)
30-m sprint (s)	4.35 ± 0.17	4.42 ± 0.24	4.48 ± 0.16	4.49 ± 0.19	3.36 (0.02)*	−0.79 (−1.35; −0.23)
*T*-test (s)	8.62 ± 0.42	8.89 ± 0.47	8.67 ± 0.46	8.78 ± 0.39	2.15 (*0.10)*	−0.38 (−0.97; 0.21)
EVO_2max_ (ml·kg·min^−1^)	49.31 ± 4.77	48.99 ± 5.92	47.30 ± 4.58	46.05 ± 4.61	2.02 (*0.12)*	0.68 (0.09; 1.27)

Q, birth quartile; ES, effect size.

^a^ Statistically different from Q1.

^b^ Statistically different from Q2.

^c^ Statistically different from Q3.

* *p* < .05.

## 4 Discussion

The aim of this study was to analyse the impact of RAEs in a representative sample of young football players in the U12, U14, and U16 categories, considering how environmental factors (e.g., age category or coaches' expectations), task-related factors (e.g., competitive level or field positions) and individual factors (e.g., the player's maturity status) influence the manifestation of RAEs. The findings of this study corroborate the prevalence of the RAEs across youth soccer categories, with significant implications for player development and talent identification. Our results align with existing literature, which demonstrates that players born earlier in the competitive year (typically those in Q1) tend to be overrepresented in youth football (39), particularly in higher competitive levels but without higher prevalence in particular positions on the field.

### 4.1 Environmental factors: age categories and coaches' efficacy expectations

Upon reviewing previous research, the findings regarding which age categories exhibit a more pronounced RAEs remain inconclusive. While it has been argued that this effect may increase during adolescence due to its interaction with maturity status, there is no unified criterion in the literature to establish that certain age categories are more susceptible to the RAEs than others (40). In this study, although we do not statistically analyze differences in the distribution of players across Q between the age categories examined, the observed trend appears to be consistent across all categories. Specifically, we found a clear overrepresentation of players born in Q1 and a notable underrepresentation of those born in Q4.

To further explore the impact of environmental variables on RAEs, we examined the efficacy expectations of coaches, which revealed a tendency to rate relatively older players more favourably in terms of physical performance potential. These expectations may contribute to the amplification of the RAEs in higher-level teams, as they likely influence selection decisions and training opportunities. Additionally, our physical performance data supported these claims, showing that relative older players, overrepresented in higher competitive levels, tend to outperformed their counterparts in physical performance tests. However, as Peña-González et al. (20) highlighted, it is necessary to determine whether players with greater RA reach higher CLs due to physical superiority prior to the selection process or if being selected for these more competitive environments leads to improved physical performance. Relatively older players, once selected, may benefit from superior training quality and intensity available at higher levels, suggesting that the RAEs becomes self-reinforcing in competitive contexts as early-born players gain access to better resources and support. To this end, it is worth considering that in this study, players with a higher RA did not exhibit better physical performance outcomes or higher efficacy expectations from coaches in the U12 category, where competitive levels do not exist, and thus, the selection process is less rigorous.

The study's findings on young football coaches' efficacy expectations reveal a nuanced impact of the RAEs on coaches' perceptions of player abilities, particularly in the U14 and U16 age categories, where the selection process becomes more pronounced due to the implementation of different competitive levels. In alignment with previous research by Peña-González et al. (10), it was found that coaches tend to have higher expectations for relatively older players, reflecting a bias favouring those born earlier in the selection year (10), further amplifying the RAEs. Such biases may stem from the perception that relatively older players possess more advanced physical or cognitive maturity, even when actual performance differences are minimal (41). This is consistent with Hancock et al.'s (26) theoretical framework, which suggests that the Pygmalion effect—the tendency for coaches' expectations to shape athlete performance—can reinforce RAEs by amplifying the advantages of early-born players through differential treatment and support (25). Coaches often interpret physical maturity as an indicator of superior potential, impacting their selection choices and leading to preferential treatment of early-born players. Our findings reinforce this notion, as the significant differences in coaches' efficacy expectations between Q1 and Q4 players in specific physical tests, particularly in the U14 category, suggest that these perceptions play a critical role in the selection and advancement of early-born athletes, often at the expense of their younger peers. This is especially relevant in the U14 category, as it marks the beginning of a more rigorous selection process due to the introduction of different competitive levels and the emergence of the greatest physical differences, as players are around the PHV stage. Moreover, this raises a broader concern regarding talent development: youth football coaches should not only consider the age group to which players belong but also their relative age when setting expectations and assessing talent. A key question is whether coaches evaluate relatively younger players based on expectations aligned with their own stage of development or in comparison to their relatively older peers within the same age category. Given that these biases can influence training opportunities and long-term development trajectories, coach education programs should emphasize the importance of assessing players based on their individual maturation patterns rather than solely on their chronological age. Addressing these biases through structured awareness and intervention strategies could foster a more inclusive approach to talent identification, where players' potential is evaluated independently of RA, as advocated by previous research aiming to support equitable youth sports development.

### 4.2 Task-related factors: competitive level and field positions

In addition to the overrepresentation of relatively older players, our study suggests that the RAEs is influenced by the competitive level in which young players participate, as higher-level teams consistently showed a more prevalent RAEs. This supports Romann et al. (17), who found that selection pressures in youth football tend to reinforce the RAEs, with a preference for athletes who demonstrate early physical and cognitive advantages, often perceived as indicators of talent (17). Expanding on our findings, the stronger RAEs observed in higher-level teams aligns with Peña-González et al. (20), who report a clear preference for young players born in the first half of the year, with early-born players making up 80.6% of high-level teams compared to only 58.5% in teams at the lowest competitive level (20). This pattern suggests that selection pressure in more competitive leagues amplifies the RAEs, likely due to coaches' inclination to associate physical and cognitive maturity with performance potential, which favours relatively older players. Findings from Götze and Hoppe (19) further reinforce this relationship, showing that RAEs is markedly more pronounced in higher leagues, especially in elite male teams, where competition intensity is greatest (19). This evidence underscores how selection pressures in top-tier competitions intensify the RAEs, further consolidating the advantages of early-born athletes in elite contexts. This supports prior research by Gutierrez Diaz Del Campo et al. (16), which demonstrated that heightened competition increases the RAEs, further favouring early-born players (16). Additionally, Peña-González et al. (20) found that while significant physical performance differences existed between competitive levels, they were not attributed solely to RA. Instead, players from higher levels outperformed those from lower levels in strength, speed, and agility tests, independent of RAEs factors. These findings suggest that the advantage of early-born players in higher levels may be reinforced by greater training quality and intensity rather than by innate physical advantages alone. Consequently, as early-born players receive superior training resources and coaching support, the RAEs becomes self-reinforcing in competitive contexts.

This study also examined the distribution of players across field positions, revealing non-significant differences in birth quartile distribution across positions compared to the expected distribution within each category, except for a reduction in RAEs among goalkeepers in the younger U12 and U14 categories. Although it has been hypothesized that certain field positions may require specific physical attributes, potentially favouring the selection of relatively older players (5), prior studies have shown no consistent RAEs differences across positions (16). However, our findings of a diminished RAEs in goalkeepers align with prior research, which often reports a less pronounced RAEs in the goalkeeper position compared to others, such as defender or forward, where older players tend to be overrepresented (42, 43). For example, Figueiredo et al. (42) observed a minimal or absent RAEs in goalkeepers within various age categories of elite Brazilian players, and similarly, Pérez-González et al. (43) found a significant RAEs in goalkeepers in only one out of four international U19 tournaments. Peña-González et al. (20) also reported a significant RAEs across all field positions (>67%) except goalkeeper (47%) when comparing halves of the birth year. These combined findings suggest that, while RAEs generally influences player selection in all field positions, the goalkeeper role may be less impacted by RA advantages, likely due to lower physical maturation demands relative to other positions (20). Similarly, the study by Romann and Fuchslocher (18) on young Swiss football players identified a stronger RAEs in defensive positions and a relatively lower effect among goalkeepers. This finding supports the hypothesis that, in positions requiring specific physical attributes, coaches may be predisposed to select relatively older players (18). It suggests that the physical development of defensive players enables them to fulfil roles demanding immediate physical performance, whereas goalkeepers—particularly at younger ages—can succeed without the same physical advantages, thereby reducing the RA bias in this position (18).

### 4.3 Individual factors: maturity status and physical performance

The findings of this study provide valuable insights into the interaction between maturation and the RAEs in youth football, while also underscoring their independence as constructs operating at distinct stages of the developmental process. The Pearson's correlation and linear regression analyses revealed a strong relationship between player's age and maturity status, particularly in the U14 and U16 categories. However, it is crucial to highlight that RAEs and maturity status, although correlated, are not interchangeable concepts. In this regard, these results are consistent with previous studies suggesting that RA advantages often align with advanced maturity status, potentially providing early-born players with additional physical and cognitive benefits that increase their likelihood of selection in competitive youth sports (1). Nevertheless, cases exist where relatively younger players demonstrate advanced maturation or relatively older players are less biologically mature than their peers (6). These distinctions underscore the complexity of the relationship between RAEs and maturity status and highlight the need to view them as interrelated but distinct factors influencing athlete development. It is important to note that differences in physical performance between players with varying RAs are not solely due to birth timing but are primarily linked to differences in the maturity status among them (12). Thus, players with a lower RA who exhibit advanced maturation also have a greater likelihood of selection (44), although this phenomenon is less common. Studies that control for the effect of maturity on performance outcomes have shown that when maturation impact is adjusted, the differences between players of different RA disappear (9, 10). This reinforces the idea that maturity status is a crucial factor in shaping selection and development opportunities for young athletes, more than just the RA (11, 12). This is particularly interesting in the process of talent identification and selection, where, despite a strong relationship between player's age and maturity status (*r* = 0.91; *R*² = 0.84), identifying outliers—cases where age and maturity status are not aligned (players with different chronological and biological ages)—may represent future success stories. By implementing training programs tailored to their maturation rather than their chronological age, these players can be effectively developed.

Differences in physical performance were observed between Q1 and Q4 players in the U14 and U16 categories. As previously suggested, these differences could be linked to the more advanced maturation of players in Q1. The greatest differences in physical performance among players with different RAs are observed in the U14 category, around the age at PHV, and where presumably larger disparities exist between players with advanced maturity status and their peers with later maturation (13, 45). Additionally, differences in speed-related variables (i.e., sprint or change of direction speed tests) are evident in the first year of the U14 category, while differences in variables related to strength among players of different quartiles emerge in the second year of this category [U14(2nd)]. This aligns with previous literature, which highlights greater increases in speed prior to PHV and more significant gains in strength once PHV is reached or surpassed, typically around the age of 14 (45–47). The findings of this study also support certain long-term development models based on maturation status, such as the YPDM (47), which emphasizes that different physical qualities develop at varying rates throughout maturation, with neural and mechanical factors playing a key role in the enhancement of speed and strength. These findings also emphasize the importance of considering the maturity status in talent identification and development processes, as relatively younger and late-maturing players may be overlooked despite their potential. Integrating maturity assessments into youth football selection could help mitigate the impact of the RAEs, promoting a fairer and more comprehensive approach to player development.

The study is not without limitations. The selected sample for this study comes from various research projects, with data collected at different points in time over the past 10 years. To mitigate this potential limitation, all assessments were conducted by the same research team using consistent materials and procedures. However, this temporal variability may still introduce inconsistencies in the data due to potential changes in selection practices and developmental trends within youth football. Additionally, a potential limitation is the possibility that some players may appear more than once in the dataset, given the longitudinal data collection over 10 years. Nevertheless, the high variability of clubs and academies involved reduces the likelihood of duplicate players, and any potential overlap does not affect the cross-sectional nature of the analyses, which treat each data point as an independent representation of the player at a specific stage of their development. Another consideration is that Tables 3, 4 present information by competitive categories, which include two-year selection groups (e.g., U14 1st and 2nd year). This could imply a loss of information by not displaying the data according to single-year categories. Although competitive categories allow players from both birth years to compete together, clubs and academies often structure their teams with players from a single birth year [e.g., only 14(1st) players competing at the highest competitive level and 14(2nd) players competing at the lower level]. Therefore, if the analysis were conducted by birth year instead of by full competitive category, it would reveal the absence of players of a specific age in certain competitive levels. The estimation of PHV in this study was conducted using the original formula by Mirwald et al. (27). While this may represent a limitation of the study, as newer formulas and corrections (e.g., Moore's correction to the Mirwald equation) have been developed in recent years (48), some of the data analyzed were collected several years ago, at a time when PHV was commonly estimated using Mirwald's formula, which was the most widely used method at that time. Regarding coaches' efficacy expectations, another limitation is that only the efficacy expectations of each coach for their own players were assessed. This means that variations in individual coaches' perceptions could lead to differences in evaluations between teams. This limitation is somewhat mitigated by including only qualified coaches with over five years of experience in youth football. Additionally, given that our sample was specific to Spanish youth football, results may differ in other cultural contexts or sports systems with varying selection pressures and competitive structures.

In terms of practical implications, addressing the RAEs require a multi-faceted approach, as recommended by Romann et al. (17). Strategies such as adjusting selection procedures, implementing birth-date banding or bio-banding, and delaying age-based competition until post-maturation could help balance developmental opportunities (49, 50). Additionally, fostering the inclusion of teams composed solely of players born in the second half of the year or late-maturing players—as parallel teams to higher-level competitive teams within the academy—could provide appropriate training stimuli tailored to their characteristics, rather than leading to early dropout from the sport. This approach could increase participation of these players in the short term, thereby acutely reducing the RAEs, while also offering the potential to identify future talent once growth and maturation processes have levelled among players. By emphasizing long-term athlete development over early success, sports organizations can foster a more equitable environment, allowing late-born athletes and those with delayed maturation to realize their potential without the disadvantage posed by early selection biases. Furthermore, considering the previously mentioned underdog hypothesis and the long-term benefits that relatively younger individuals may develop, it is worth exploring whether players should be challenged by training and competing with older individuals as part of their development. A more flexible and dynamic youth development pathway, in which players have opportunities to train and compete across various age groups, could be beneficial for all athletes.

Our study confirms that RAEs remain a significant factor in youth football, particularly within competitive structures that favor early physical and cognitive advantages. By analyzing a representative sample across different age categories (U12, U14, and U16) recruited over a 10-year period, our findings provide a comprehensive perspective on how environmental (age category, coaches' expectations), task-related (competitive level, field position), and individual (maturity status, physical performance) factors contribute to RAEs. The consistent overrepresentation of early-born players, especially in higher competitive levels, highlights that selection processes continue to favor those with early physical advantages, reinforcing systemic biases in talent identification. Coaches' expectations further amplify this effect, as they often perceive relatively older players as having greater potential, influencing their selection and development opportunities. However, our study reinforces that RAEs and maturity status, though related, are distinct constructs, emphasizing the need for talent identification models that prioritize biological rather than chronological age. Educating coaches to recognize and address these biases is also crucial in fostering a more inclusive talent development framework that prioritizes long-term potential over short-term success.

## Data Availability

The data supporting this research will be available upon request via email to the corresponding author from the time of publication and for the following three years, provided that a reasonable justification.
